# The complete mitochondrial genome sequence of *Cletus punctiger* (Heteroptera: Coreidae)

**DOI:** 10.1080/23802359.2019.1674732

**Published:** 2019-10-09

**Authors:** Huanyu Zhang, Xiaolei Yu, Weiling Jiang, Han Gao, Wei Tan, Wenxiu Wang, Yuxia Liu, Xiaoxuan Tian

**Affiliations:** Tianjin State Key Laboratory of Modern Chinese Medicine, Tianjin University of Traditional Chinese Medicine, Tianjin, P. R. China

**Keywords:** *Cletus punctiger*, mitogenome, phylogeny

## Abstract

*Cletus punctiger* is a famous economic crop pest in China, especially for rice. Here, we first reported the complete mitochondrial genome sequence of this pest. The mitochondrial genome is 16,166 bp in length, including 13 protein-coding genes (PCGs), 22 tRNA genes, 2 rRNA genes, and 1 control region (D-Loop). The maximum likelihood (ML) phylogenetic tree confirmed that *C. punctiger* belonged to the Coreidae subfamily.

*Cletus punctiger* is an important pest of rice, which often sucks the juice of young panicle, causing a decline in rice yield. This pest mainly appears from mid-July to early August and widely distributed throughout China, especially in the south of the Yangtze River. To get a deeper understanding of the phylogenetic relationship of this pest, we sequenced and reported its complete mitochondrial genome.

The specimen was collected from Nanchang city, Jiangxi province (115.85°E, 28.68°N), China. The genomic DNA of *C. punctiger* was extracted using a Genomic DNA Extraction Kit (Shanghai, China), following the manufacturer’s instructions. The sample was stored at the Tianjin State Key Laboratory of Modern Chinese Medicine (voucher number: DCYC-1). Total DNA was sequenced using the Illumina Hiseq X Ten platform (Illumina, San Diego, CA, USA). The complete mitogenome of *C. punctiger* was assembled using NOVOPlasty version 3.1 (Dierckxsens et al. 2017), and the *Cloresmus pulchellus* (Accession number: MF497719) was used as reference (Dierckxsens et al. 2017). The mitochondrial genome of *C. punctiger* was annotated using the MITOS web server (Bernt et al. 2013). Then, the annotated genome was inspected manually. The tRNA genes were confirmed using the tRNAscan-SE search server (Lowe and Eddy 1997).

The complete mitogenome of *C. punctiger* is 16,166 bp in length, consisting of 13 protein-coding genes (PCGs), 22 tRNA genes, 2 rRNA genes, and 1 control region. The composition of the mitogenome is 41.9% A, 15.9% C, 10.7% G, 31.5% T, and with 26.6% guanine-cytosine (GC) content. It is similar to other insect mitogenomes (Gao et al. 2019; Yu et al. 2019).

There are four genes (*ND2*, *ATP6*, *COIII*, and *CytB*) starting with ATG initiation codon, three PCGs with ATT codon (*COII*, *ND3*, and *ND4L*), two PCGs (*ND5* and *ND4*) initiation codons are TAC, and the remaining genes *ATP8, ND6, ND1,* and *COI* are starting with ATC, ATA, TAA, and TTG codons, respectively. Correspondingly, 7 PCGs (*ND2, ATP8, ATP6, COIII, ND3, ND4L,* and *ND6*) share the stop codon TAA. *CytB* and *ND1* are terminated with TAG and ATC, respectively. While *COI* and *COII* genes are terminated with an incomplete stop codon (T-). Two PCGs (*ND5* and *ND4*) are terminated with an incomplete stop codon (A-). A maximum likelihood (ML) phylogenetic tree was constructed based on the complete mitochondrial genome from NCBI database (Figure 1), with *Tachyta nana* as an outgroup. The result indicated that *C. punctiger* formed an independent lineage in the Coreinae subfamily. Overall, the complete mitochondrial genome of *C. punctiger* can contribute to further phylogenetic study within *Coreidae*.

**Figure 1.**
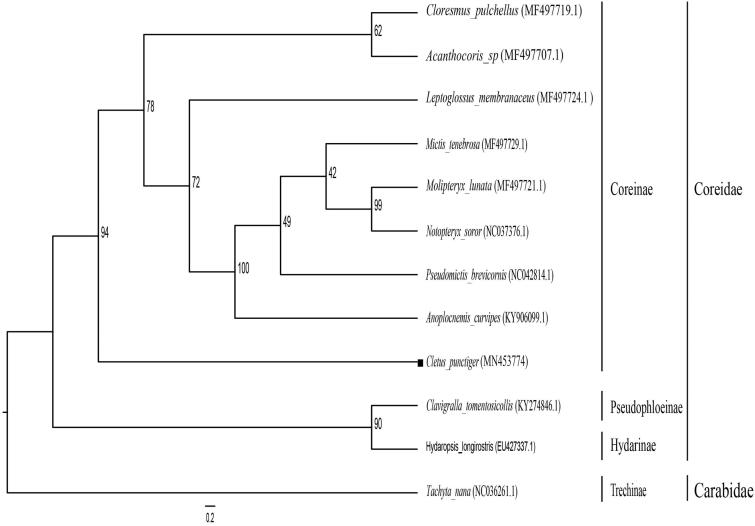
Maximum likelihood phylogenetic tree of *Cletus punctiger* constructed with 12 species. Numbers at nodes are values for bootstrap support.
